# Latent TB infection in solid organ transplant recipients in Serbia: A single center experience from a low TB burden country

**DOI:** 10.1371/journal.pone.0339925

**Published:** 2026-01-22

**Authors:** Jovana Milisavljevic, Maja Stosic, Nataša Jovanovic, Tatjana Adzic Vukicevic

**Affiliations:** 1 Primary Health Center “Zvezdara”, Belgrade, Serbia; 2 Institute of Public Health of Serbia “Dr Milan Jovanovic Batut”, Belgrade, Serbia; 3 Singidunum University, Belgrade, Serbia; 4 Nephrology Clinic of the University Clinical Center of Serbia, Belgrade, Serbia; 5 Clinic for Pulmonology, University Clinical Center of Serbia, Belgrade, Serbia; 6 School of Medicine, University of Belgrade, Belgrade, Serbia; St Petersburg Pasteur Institute, RUSSIAN FEDERATION

## Abstract

**Background:**

Solid organ transplant (SOT) recipients face a heightened risk of latent tuberculosis infection (LTBI) reactivation and progression to active tuberculosis (TB) because of their immunosuppressed state**.** In low TB burden countries like Serbia, data on LTBI and TB incidence in transplant recipients are limited.

**Objectives:**

To determine the prevalence of latent tuberculosis infection and the incidence of active tuberculosis among kidney and liver transplant recipients, and to identify factors associated with LTBI in this high-risk group.

**Methods:**

A retrospective cohort study was conducted among 275 adult SOT recipients (203 kidney, 72 liver) who underwent transplantation at a tertiary care center in Serbia between 2007 and 2017. LTBI was assessed using Interferon-Gamma Release Assay. Patients were followed for five years post-transplantation to monitor for LTBI and active TB. Multivariable logistic regression was used to identify independent predictors of LTBI.

**Results:**

The post-transplant LTBI prevalence among kidney transplant recipients was 23.4 (95% CI = 14.7%–35.1%), while 5.6 (95% CI = 2.2%–13.4%) among liver transplant recipients (p = 0.003). The crude incidence of post-transplant TB was 151.4 per 100,000 person-years, over seven times higher than the general population in Serbia. Median time to TB diagnosis was 6 months (IQR: 3.0–12.0). Independent predictor of LTBI was previous contact with a TB case (OR =26.08; 95% CI = 5.63–120.90).

**Conclusions:**

The substantially elevated burden of LTBI and TB among SOT recipients underscores the need for systematic LTBI screening and targeted preventive treatment. Strengthened collaboration among transplant physicians, pulmonologists, and public health teams is essential to improve TB prevention strategies in this vulnerable population, particularly in countries aiming for TB elimination.

## Introduction

Tuberculosis (TB) remains the most prevalent infectious disease globally, excluding coronavirus disease 2019 (COVID-19) during the pandemic years 2019–2023. In 2023, 10.8 million people developed TB, with 8.2 million new cases reported—marking the highest number ever recorded [1]. Recent estimates indicate that approximately 1.7 billion people worldwide are latently infected with *Mycobacterium tuberculosis*. Latent tuberculosis infection (LTBI) is defined as a sustained immune response to *M. tuberculosis* antigens without clinical or radiological evidence of active TB disease. It is estimated that up to 10% of individuals with LTBI may progress to active TB over their lifetime [2]. The risk of progression to active TB is highest among individuals with immunosuppressive conditions, such as diabetes mellitus, malnutrition, HIV infection, or those undergoing treatment with immunosuppressive or biological agents, such as organ transplant recipients [3]. Solid organ transplantation (SOT)—most commonly kidney and liver transplantation—is the standard treatment for patients with end-stage renal or hepatic disease [4]. However, post-transplant infections pose a major challenge to both graft survival and patient outcomes. Among these infections, TB remains one of the most significant threats. The prevalence of active TB in SOT recipients ranges from 0.3% to 6.4%, and can be as high as 15.2% in countries with a high TB burden. The incidence of post-transplant TB in SOT recipients is 20–74 times higher than in the general population [5]. In most cases, active TB following transplantation results from reactivation of latent infection. Multiple risk factors predispose kidney and liver transplant recipients to developing TB at higher rates than the general population. Transplant-related risk factors include immunosuppressive therapies such as T-cell depleting agents (e.g., anti-thymocyte globulin), cytotoxic T-lymphocyte-associated protein-4 inhibitors (e.g., belatacept), calcineurin inhibitors (tacrolimus, cyclosporine), anti-metabolites (mycophenolate mofetil, azathioprine), and corticosteroids [6]. Chronic graft dysfunction and rejection—requiring intensified immunosuppression—also increase the risk. Recipient-related factors that influence the risk of developing active TB or reactivating LTBI include older age, male sex, smoking, malnutrition, diabetes mellitus (DM), chronic obstructive pulmonary disease (COPD), latent TB before transplantation, chronic liver disease, hepatitis C virus infection, opportunistic infections (e.g., cytomegalovirus, *Pneumocystis jirovecii*, *Nocardia*), autoimmune diseases, and long-term hemodialysis [7]. Donor-related factors that influence the risk of developing active TB or reactivating LTBI include cadaveric transplantation and various social and medical conditions, such as homelessness, smoking, alcohol abuse, known TB exposure, diabetes mellitus, a body mass index below 18.5 kg/m², and a history of untreated TB [8]. Additionally, the TB burden of the recipient’s country significantly influences post-transplant TB incidence. Serbia is classified as a low TB burden country, with a reported incidence rate of 7.1 per 100,000 population and estimated prevalence of 13,7% [9,10]. While solid organ transplantation has been performed in Serbia since 1973, national LTBI screening guidelines were developed in 2019, not particular for SOT recipients. However, based on the expert opinions, there were certain clinical protocols for LTBI screening among transplanted patients established long before 2019, unfortunately not publicly available, but routine monitoring of coverage of pre-transplant and post-transplant TB screening has not been implemented so far. The aim of this study was to assess the prevalence of LTBI and the incidence of active TB among kidney and liver transplant recipients, and to identify factors associated with LTBI in this high-risk population.

## Materials and methods

### Study design

We conducted a retrospective cohort study using data from patients who underwent kidney or liver transplantation at the Center for Organ Transplantation of the Emergency Center, University Clinical Center of Serbia, from January 1, 2007, to December 31, 2017. Patients were followed for a five-year period to monitor LTBI and development of active TB.

### Data collection and procedure

A total of 275 consecutive patients were included. Medical records were reviewed to collect demographic and socio-economic information, history of prior TB, previous contact with TB cases, smoking status, alcohol use, comorbidities, type of transplantation, results of post-transplant LTBI testing using Interferon-Gamma Release Assay (IGRA) -” QuantiFERON TB Gold“t est, number of years after transplantation, and any occurrence of active TB during follow-up.

Data were accessed for research purposes between January 1 and January 30, 2023. The authors did not have access to any personally identifiable patient information during or after data collection.

Based on above mentioned clinical protocol for LTBI screening, all study participants should be clinically evaluated and screened for LTBI regardless of perceived exposure risk. In addition, LTBI testing should be performed before and after transplantation. However, during the review of medical records, there was no data in relation to pre-transplant LTBI testing at all. Therefore, we presented only the results of post-transplant LTBI testing. Exclusion of active TB disease was performed prior to targeted LTBI testing, implemented every six months after transplantation. IGRA testing was performed to all LTBI tested study participants. Repeat testing were used when initial results were indeterminate. Patients with previous TB treatment history was clinically evaluated and active TB disease was excluded prior to LTBI testing.

### Inclusion and exclusion criteria

Inclusion criteria were adult kidney or liver transplant recipients aged ≥18 years.

### Statistical analysis

Descriptive statistics were used to summarize the data. Categorical variables were expressed as frequencies and percentages.

Univariate logistic regression analysis (ULRA) was conducted to identify variables potentially associated with LTBI (p < 0.05). Variables found to be significant were then entered into a multivariable logistic regression analysis (MLRA). A p-value < 0.05 was considered statistically significant. All statistical analyses were performed using IBM SPSS Statistics, version 22 (IBM Corp., Armonk, NY, USA).

### Ethical approval

The study was approved by the Ethical Board of the Clinical Center of Serbia (Approval No. 301/1) and the Ethics Review Committee of the School of Medicine, University of Belgrade (Approval No. 1322/XI-1). Consent for publication of raw data obtained from study participants. The research team signed a confidentiality agreement.

## Results

Out of 275 patients included in the study, 165 (60%) were male. The mean age for all participants was 52.5 ± 13.1 years. Almost half of all study participants, 136 (49.5%), were tested for LTBI after transplantation. Other characteristics of all study participants are presented in Table 1.

**Table 1:** Characteristics of the study participants (n = 275).

Characteristics	n	%
**Sex**		
Male	165	60.0
Female	110	40.0
Age (years) mean, SD	52.5 ± 13.1	
**Employment**		
Employed	160	58.2
Student	17	6.2
Unemployed	98	35.5
**Residence**		
Urban	70	25.5
Rural	205	74.5
**Smoking status**		
Current smoking	80	29.1
Non-smoking	195	70.9
**Alcohol consumption**		
Yes	47	17.1
No	228	82.9
**Previous contact with TB case**	16	5.8
**Previous TB disease**	1	0.4
**Concomitant diseases**		
Cardiac (angina pectoris, ischemic cardiomyopathy)	77	28.0
Arterial hypertension	227	82.5
Diabetes	60	21.8
At least one co-morbidity	228	82.9
**Type of transplantation**		
Kidney	203	73.8
Liver	72	26.2
LTBI tested after transplantation	136	49.5
Number of years after transplantation	9.6 ± 3.4	
Diagnosed active TB disease	4	1.5

Among LTBI tested study participants, 117 (86.0%) were LTBI negative while 19 ((14.0%) were LTBI positive. The mean age was 53.2 ± 12.9 years in LTBI negative group and 48.2 ± 14.7 years in LTBI positive group. Males accounted for 62.4% in LTBI negative group and 52.3% in LTBI positive group. Among LTBI tested, 64 (31.5%) were kidney transplant recipients, while 72 (100.0%) were liver transplant recipients. Among LTBI tested kidney transplant recipients, 15 were LTBI positive, yielding to a prevalence value of 23.4 (95% CI = 14.7%–35.1%). Among LTBI tested liver transplant recipients, 4 were LTBI positive yielding to a prevalence value of 5.6 (95% CI = 2.2%–13.4%). There is a statistically significant difference between the prevalence of LTBI among kidney and liver transplant recipients (p = 0.003).

The primary outcome measure was the crude incidence rate of post-transplant tuberculosis (TB). Incidence rate was calculated per 100,000 person-years, with corresponding 95% confidence intervals (CI). To account for variations in immunosuppression burden, incidence rates were stratified by time periods during and after the first-year post-transplant, as TB risk is hypothesized to be highest in the early months following transplantation [11]. Over a total follow-up time of 2,642 person-years, 4 out of 275 recipients (1.45%) were diagnosed with incident TB. This yielded an overall crude incidence rate of 151.4 cases per 100,000 person-years. The median time to TB diagnosis among the four cases was 6 months (interquartile range [IQR] 3.0–12.0 months). No deaths occurred during the follow-up period.

Bivariate analysis revealed no significant differences between the LTBI negative and LTBI positive groups regarding sex, age, employment status, residence, previous TB disease, comorbidities and alcohol consumption.

However, there were statistically significant differences in smoking status (Odds Ratio [OR] = 0.22; 95% Confidence Interval [CI] = 0.05–0.99), previous contact with TB case (OR = 30.80; 95% CI = 8.60–110.50) and type of transplantation (OR = 0.19; 95% CI = 0.06–0.61) – Table 2.

**Table 2:** Results of univariate logistic regression analysis (dependent variable is latent TB infection).

Characteristics	Total (n = 136)		LTBI negative (n = 117)		LTBI positive (n = 19)		OR (95% CI)	p value
	n	%	n	%	n	%		
**Sex**								
Male	83	61.0	73	62.4	10	52.3	ref	0.420
Female	53	39.0	44	37.6	9	47.4	1.49 (0.56- 3.96)	
Age (years) mean, SD	52.5 ± 13.2		53.2 ± 12.9		48.2 ± 14.7		0.97 (0.94- 1.01)	0.127
**Employment**								
Employed	83	61.0	72	61.5	11	57.9	ref	
Student	11	8.1	10	29.9	1	5.3	0.66 (0.08- 5.63)	0.699
Unemployed	42	30.9	35	8.5	7	36.8	1.31 (0.47- 3.67)	0.608
**Residence**								
Urban	103	75.7	90	76.9	13	68.4	ref	0.425
Rural	33	24.3	27	23.1	6	31.6	0.65 (0.23- 1.87)	
**Smoking status**								
Current smoking	41	30.1	39	33.3	2	10.5	ref	**0.049**
Non-smoking	95	69.9	78	66.7	17	89.5	0.22 (0.05- 0.99)	
**Alcohol consumption**								
Yes	36	26.5	31	26.5	5	26.3	ref	
No	100	73.5	86	73.5	14	73.7	0.99 (0.33- 2.98)	0.987
**Previous contact with TB case**	16	11.8	5	4.3	11	57.9	30.80 (8.60- 110.50)	**0.000**
**Previous TB disease**	1	0.7	1	0.9	0	0.0	n/a	1.000
**Concomitant diseases**								
Cardiac (angina pectoris, ischemic cardiomyopathy)	34	25.0	31	26.5	3	16.8	0.52 (0.14- 1.91)	0.324
Arterial hypertension	97	71.3	80	68.4	17	89.5	3.93 (0.86- 17.90)	0.077
Diabetes	36	26.5	32	27.4	4	21.1	0.71 (0.22- 2.30)	0.565
At least one co-morbidity	97	71.3	80	68.4	17	89.5	3.93 (0.86- 17.90)	0.077
**Type of transplantation**								
Kidney	64	47.1	49	41.9	15	78.9	ref	
Liver	72	52.9	68	58.1	4	21.1	0.19 (0.06- 0.61)	**0.005**
Number of years after transplantation	7.2 ± 2.4		7.3 ± 2.4		6.6 ± 2.0		0.88 (0.70- 1.11)	0.266
Diagnosed active TB disease	2	1.5	2	1.7	0	0.0	n/a	1.000

Variables found to be significant at p < 0.05 (smoking status, previous contact with TB case and type of transplantation) were entered into a multivariable logistic regression analysis.

In the multivariable logistic regression analysis, one factor was independently associated with latent TB infection among study participants: previous contact with a TB case (OR = 26.08; 95% CI = 5.63–120.90), -Table 3.

**Table 3:** Results of multivariable logistic regression analysis (dependent variable is latent TB infection).

Variable	OR (95% CI)	p-value
Previous contact with TB case	26.08 (5.63-120.90)	0.000
Smoking status	0.22 (0.04- 1.25)	0.087
Type of transplantation	0.73 (0.17-3.01)	0.666

The multivariable model was statistically significant and accounted for approximately 42% of the variance in LTBI, as indicated by the model’s goodness-of-fit statistics.

## Discussion

In this study, we evaluated 275 solid organ transplant recipients—kidney and liver. The majority were kidney transplant recipients (73.8%), reflecting the higher frequency of renal transplantations in our center. Males predominated among all study participants (60.0%). Among LTBI tested study participants, mean age was 532.29 ± 12.9 years in LTBI negative group and 48.2 ± 14.7 years in LTBI positive group. Males accounted for 620.45% in LTBI negative group and 52.36% in LTBI positive study participants. These findings are comparable to a previous review conducted between January 2015 and December 2018, which included 766 SOT recipients and found a median age of 43.8 ± 13.7 years, predominantly male, with kidney transplants being the most common. Heart and kidney transplant recipients were the youngest in that cohort [12].

Our findings indicate that the crude incidence rate of post-transplant TB was 151.4 per 100,000 person-years, over seven times higher than the general population of Serbia and more than five times higher than reported rates in England, Wales, and Northern Ireland [11]. This highlights the urgent need to strengthen TB prevention strategies in transplant populations.

The post-transplant LTBI prevalence among kidney transplant recipients was 23.4 (95% CI = 14.7%–35.1%), while 5.6 (95% CI = 2.2%–13.4%) among liver transplant recipients. These rates are consistent with other studies conducted in low TB burden countries. Kidney transplant recipients may have a higher prevalence of LTBI for several reasons. First, chronic kidney disease (CKD) and long-term dialysis are known risk factors for LTBI due to immune dysfunction and frequent healthcare exposure. Second, regional epidemiology and socioeconomic factors among patients with end-stage renal disease (ESRD) might increase baseline exposure to *Mycobacterium tuberculosis*. Finally, pre-transplant screening practices for LTBI may differ across organ types, leading to higher detection rates among kidney recipients. [5,13]. However, true LTBI prevalence is likely underestimated, particularly in the renal group, due to suboptimal testing coverage. Notably, kidney transplant recipients were significantly more likely to be tested positive for LTBI when screened, despite being tested less frequently than liver transplant recipients.

Prior contact with a person with active TB was very strong independent predictor of LTBI in our cohort, consistent with global evidence identifying close contact as the primary determinant for both LTBI and progression to active disease [13]. The risk of LTBI progressing to active TB varies by bacterial, environmental, and host factors. Among host-related risk factors, aging, male sex, genetic predisposition, immunosuppression, diabetes, malnutrition, anemia, pregnancy, HIV infection, and chronic diseases—including end-stage renal disease and post-transplantation—are significant contributors [14–16].

We did not find that having at least one comorbidity was independently associated with LTBI which is different from the results of other studies. Namely, hypertension, cardiac diseases (angina pectoris and ischemic cardiomyopathy), and diabetes mellitus were the most frequently reported comorbidities, particularly among kidney transplant recipients. This may be explained by end-stage renal disease and its association with nephro-angiosclerosis and cardiovascular complications. In addition, uremia impairs cell-mediated immunity via T-cell dysfunction, reduced cytokine production, and macrophage impairment → higher risk of TB reactivation and false-negative TST/IGRA [17,18]. Cirrhotic immune dysfunction (defective macrophage/Kupffer cell activity, complement deficiency, T-cell exhaustion) reduces containment of *M. tuberculosis* and increases false-negative test results [19]. Diabetes, in particular, is a known risk factor for both LTBI and active TB, likely due to impaired immune function [20]. Hyperglycemia impairs macrophage and neutrophil function, decreases IFN-γ and IL-12 signaling, and blunts Th1 responses leading to 2–3-fold higher reactivation risk [21–23].

Interestingly, chronic obstructive pulmonary disease (COPD) was not reported among our participants, likely due to the relatively low prevalence of current smokers among study participants (29.1%). In contrast, other studies have reported a strong association between TB and COPD, with post-TB sequelae often contributing to long-term pulmonary impairment, [24]. Another explanation for low COPD in this study would be because of the participants included (liver or kidney transplant). Usually, lung transplant candidates have more COPD vs other solid organ transplant candidates whose respiratory status should be usual optimal to be a candidate for transplant [25]. IGRA positivity in COPD patients has been linked to cumulative cigarette exposure and corticosteroid use [26], which were not prominent in our cohort.

Our results also showed that the median time to TB diagnosis was 6 months, supporting findings from other studies showing that the majority of post-transplant TB cases develop within the first year, particularly within a median of 238 days [27]. Although kidney transplantation improves survival, it also increases susceptibility to infections due to immunosuppressive therapy [28,29].

We found low percentage of alcohol consumers among study participants that differs from previous findings that attribute liver transplantation to alcohol-related cirrhosis that impairs immune defense mechanisms, particularly alveolar macrophage function, thereby increasing susceptibility to *Mycobacterium tuberculosis* infection [30].

According to the latest WHO recommendations, LTBI screening should be conducted using tuberculin skin testing (TST) or interferon-gamma release assays (IGRAs). These tests are particularly recommended in high-risk groups such as SOT recipients, regardless of national TB burden. In our study, IGRA was the only test used and was administered to almost one-half of study participants—a pattern similar to other countries [12]. Testing rates were significantly higher among liver recipients (100.0%) compared to kidney recipients (31.5%). Guidelines vary in their approach, with some recommending IGRA alone, and others endorsing a two-step approach beginning with TST. False-positive TST results may occur due to prior BCG vaccination or exposure to non-tuberculous mycobacteria. Therefore, IGRA test is highly recommended [31,32].

A positive TST or IGRA indicates LTBI, but a negative result does not exclude it, particularly in immunocompromised populations. Therefore, comprehensive pre-transplant screening—encompassing detailed epidemiologic history, imaging, and IGRA testing—is essential [33]. Studies have shown that individuals with a positive IGRA result have a 2.1-fold higher risk of developing active TB compared to those with a negative result [34]. Additionally, IGRA positivity in SOT recipients has been associated with previous TB history, known TB exposure, and radiological evidence of sequelae [35].

This study has several limitations. The relatively small sample size and low number of TB cases limited our ability to perform multivariable regression analysis for active TB. The low coverage of LTBI testing among renal transplant recipients’ groups further constrained our findings. In addition, due to unavailability of medical records data, we could not analyze pre-transplant LTBI testing coverage and results. Moreover, due to absence of publicly available clinical protocol for organ transplantation and medical records data, we could not evaluate the associations between immunosuppressive drugs used and LTBI. Despite these limitations, this is, to our knowledge, the first study in Serbia and the Balkan region to examine both LTBI and active TB among SOT recipients. Our findings provide preliminary insight into potential predictors of LTBI and underscore the need for further more detailed research with larger cohorts.

## Conclusions

Our findings reveal a substantially higher burden of LTBI among solid organ transplant recipients compared to the general population, emphasizing the urgent need for standardized, systematic LTBI screening and tuberculosis preventive treatment, which is currently lacking. In countries with low TB incidence that aim for TB elimination, enhanced collaboration between transplant physicians, pulmonologists, and public health professionals is essential. Strengthening TB prevention strategies in high-risk groups such as SOT recipients should be a key component of national TB control programs.

## Supporting information

S1 File Database. (XLSX)
